# Influence of coupled water and thermal treatments on the fracture characteristics of a typical sandstone

**DOI:** 10.1038/s41598-024-62025-y

**Published:** 2024-05-17

**Authors:** Yi Luo, Haohong Zhong, Li Ren, Cunbao Li

**Affiliations:** 1 School of Environment and Civil Engineering, Dongguan University of Technology, Dongguan, 523808 China; 2 Guangdong Provincial Key Laboratory of Intelligent Disaster Prevention and Emergency Technologies for Urban Lifeline Engineering, Dongguan, 523808 China; 3 Key Laboratory of Deep Underground Science and Engineering (MOE), College of Architecture and Environment, Sichuan University, Chengdu, 610065 China; 4 State Key Laboratory of Intelligent Construction and Healthy Operation and Maintenance of Deep Underground Engineering, Shenzhen University, Shenzhen, 518060 China

**Keywords:** Notched deep beam specimen, Fracture toughness, Pure mode I, Pure mode II, Water, Thermal, Civil engineering, Geothermal energy, Fossil fuels, Petrology

## Abstract

Understanding the fracture behavior of rock after coupled water and thermal environment is important for many geotechnical projects. This study examines the influence of coupled water and thermal treatments on the fracture toughness and characteristics of a typical sandstone under mode I and mode II loading conditions. Notched deep beam (NDB) specimens were utilized and subjected to soaking treatments at various water temperatures (23 °C, 60 °C, and 99 °C). The experimental results indicate a significant reduction in both mode I and mode II fracture toughness values, with reductions ranging from 15.4% to 13.2% for mode I and 26.1% to 8.9% for mode II respectively. As the water temperatures increase, a slightly rising trend is observed in both mode I and mode II fracture toughness within the examined temperature range. Sandstone specimens displayed typical brittle fracture characteristics at lower soaking temperatures. For mode I specimens, an increase in ductility was evident with higher soaking temperatures, while the ductile behavior is less pronounced in the mode II specimens. Based on the Maximum Tangential Stress (MTS) criterion and the Generalized Maximum Tangential Stress (GMTS) criterion, the predicted values of mode II fracture toughness and the fracture process zone (FPZ) were discussed. The results show that both the GMTS and MTS criteria exhibit inaccuracies in predicting the mode II fracture toughness of sandstone treated at different soaking water temperatures. However, the GMTS criterion, which incorporates *T*-stress, demonstrates smaller errors compared to the MTS criterion. The study shows that the radius *r*_0_ of the fracture process zone is not a constant under both mode I and mode II loading conditions. The calculation of the fracture process zone radius* r*_0_ in the GMTS criterion requires further theoretical and experimental study.

## Introduction

Natural rocks contain various initial microcracks, fissures, and other flaws. The deformation and failure process of rock often involves the initiation, expansion, coalescence, and propagation of these internal cracks^1^. Fractures are a common occurrence in various geological engineering activities such as mining, tunnel excavation, geothermal development, and unconventional oil and gas extraction. For instance, in deep tight sandstone gas extraction, hydraulic fracturing techniques are frequently employed to induce fractures in the rock^2,3^. In practical engineering applications, rock masses often exist under conditions of high temperature and humidity. For example, in projects involving slopes and dams, the rock mass is exposed to an environment saturated with groundwater or other fluids^4,5^. During the development of hot dry rock geothermal energy, rock layers are subjected to the effects of high temperature and fluid saturation, resulting in mechanical properties that differ from those in a dry state^6^. Consequently, understanding the fracture characteristics of rocks in a coupled water and thermal environment is of significant practical importance for the effective execution of many geotechnical projects.

The fracture toughness of rock characterizes its ability to resist crack initiation and propagation, representing a crucial mechanical parameter in determining rock mechanical properties and in the design and analysis of rock structures^7^. Based on different loading modes, cracks are classified as mode I (opening mode), mode II (sliding mode), and mode III (tearing mode). The mode I fracture and mode II fracture are common in rock engineering^8^, thus the mode I and mode II fracture toughness as key parameters have garnered widespread attention in research on rock fracture characteristics^9–11^. Currently, scholars have conducted extensive research on the physical and mechanical properties such as compressive strength, shear strength and elastic modulus of rocks under water–rock interaction^12,13^. These findings indicate that the physical and mechanical properties of rocks undergo varying degrees of degradation due to the water, and the extent of this weakening is closely related to factors such as the type of rock, and the soaking solution. Several researchers have investigated the impact of varying degrees of water saturation on the tensile strength and fracture toughness of rocks^14–19^. They have found that water significantly softens rock properties, and both the tensile strength and fracture toughness of rocks decrease progressively with increasing water saturation. Cai et al.^20^ conducted research on the failure modes and weakening mechanisms of sandstone subjected to water soaking. The study found that water made the rocks more ductile and the extent of sandstone weakening is related to the rock's porosity and mineral composition. The failure mode of sandstone often shifts from a tensile mode in dry conditions to a shear-dominated mode under wet conditions. Some researchers have observed that dry–wet cycling affects the physical properties of sandstone, such as density, P-wave velocity, and porosity^21,22^ . Huang et al.^21^ found that dry–wet cycles reduced the uniaxial compressive strength of sandstone and increased its water absorption and porosity. Cai et al.^22^ demonstrated that with the increase in the number of dry–wet cycles, the density and average longitudinal wave velocity of the sandstone decreased, while its porosity gradually increased. After 10, 20, 30, 40, and 50 dry–wet cycles, the density decreased by 2.16%, 3.86%, 4.38%, 4.80%, and 5.38%, respectively. Dehestani et al.^23,24^ conducted experiments on mode I, mode II and mixed-mode (I + II) fracture in sandstone under dry–wet cycling. Their research revealed that dry–wet cycling degrades the fracture toughness of sandstone, with this degradation becoming more pronounced with an increasing number of cycles.

In recent years, numerous scholars have studied the impact of high temperatures on the physical, mechanical properties, and fracture parameters of rocks^25^. Pathiranagei et al.^26^ observed that high temperatures ranging from 200 to 400℃ impact rock properties; notably, porosity decreases as rock strength increases. However, at temperatures ≥ 600 °C, there is a significant increase in rock porosity and a decrease in rock strength. Research indicates that there is a certain threshold for the physical and mechanical properties and fracture characteristics of rocks under the influence of temperature. Some researchers discovered that the tensile strength and uniaxial compressive strength of rock samples exhibit a threshold, initially increasing and then decreasing with rising temperatures^27–29^. Some studies have shown that the fracturing behavior of rocks varies with temperature^30–32^. The fracture toughness of rocks gradually increases until it reaches a threshold, beyond which it tends to decrease as temperatures rise^33,34^. Experiments conducted by Feng et al.^33^ and Roy et al.^35^ determined that the temperature threshold for sandstone is approximately 100 °C and 200 °C, respectively. As thermal treatment temperatures gradually increase, the fracture characteristic of granite shifts from brittle to ductile^36,37^. When the temperature exceeds a specific threshold, a transition from brittle to ductile behavior occurs^38^. Alzo’ubi et al.^39^ and Alneasan et al.^40^ have determined that this threshold is 500 °C for granite. Below 500 °C, the brittleness of granite increases with rising temperatures; while above 500 °C, the increase in temperature leads to enhanced ductility in the granite. The impact of temperature on the density of sandstone is far greater than on granite, while the effect on longitudinal wave velocity is the opposite^41,42^. Kang et al.^43^ conducted experiments on mode I, mode II and mixed- mode I/II fracture in sandstone treated at different temperatures, indicating that the temperature range that affects rock fracture toughness varies under different loading modes. Zhang et al.^44^ compared the mode I fracture toughness of granite subjected to thermal treatments with those under coupled thermo-hydro-mechanical treatments and found fluctuations in the fracture toughness of granite subjected to thermo-hydro-mechanical treatments. However, they primarily focus on the mode I fracture toughness of rock materials, with a lack of studies on the effects of high temperature and fluid saturation environments on both mode I and mode II fracture characteristics of rocks.

In summary, while numerous studies have focused on the individual effects of water or temperatures on rocks mechanical properties, studies on the rocks fracture behavior under coupled water and thermal environments are limited. To address this shortcoming, this study conducts a series of fracture toughness tests using notched deep beam (NDB) specimens of sandstone. These specimens were subjected to soaking in water at varying temperatures (23 °C, 60 °C, and 99 °C) to simulate coupled water and thermal environments. The study provides a comprehensive examination of the influence of coupled water and thermal treatments on both mode I and mode II fracture characteristics of sandstone. Additionally, the study predicts the mode II fracture toughness of rock under various conditions based on the Maximum Tangential Stress criterion and discusses the calculation of the fracture process zone for pure mode I and mode II loading.

## Experimental methods

### Stress intensity factors for the NDB specimen

**Figure 1:**
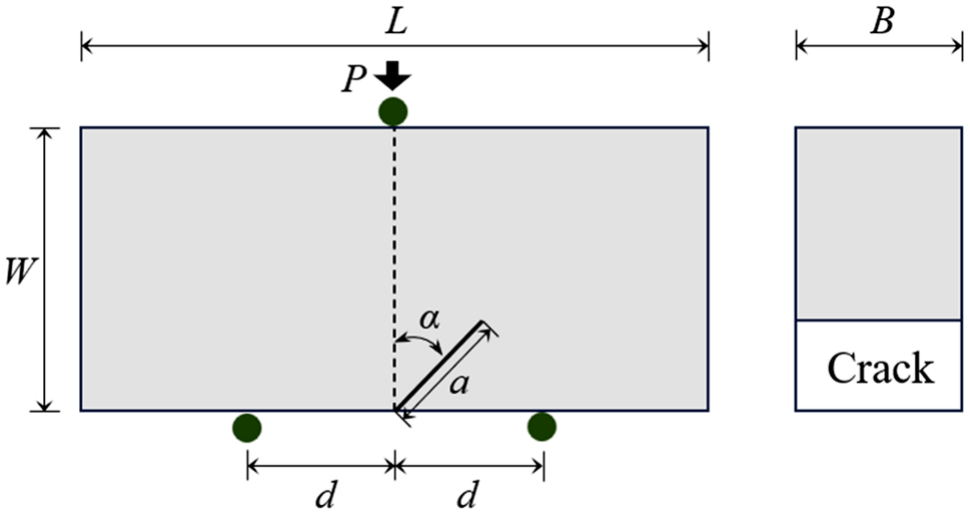
Diagram of the NDB specimen under three-point bending.

The configuration of the NDB specimen^45^ is a brick-type rectangle with a length-to-width ratio (*L/W*) of 2.0, featuring a single-edge crack of length *a*, as depicted in Fig. 1. The NDB samples can provide pure mode I, pure mode II, and any intermediate mixed-mode loading conditions by adjusting the crack angle (*α*), crack length (*a*), and the span (2*d*) between the two bottom supports under symmetric three-point bending conditions. In contrast to the cracked chevron-notched Brazilian disk (CCNBD) and semicircular bend (SCB) specimens recommended by International Society for Rock Mechanics (ISRM), NDB specimens with any size can be easily obtained by cutting from rock blocks to study the fracture behavior^46–48^. The loading equipment required for NDB specimens is straightforward. Therefore, NDB specimens with two different crack angles made of sandstone are used in this study to investigate the pure mode I and mode II fracture toughness of sandstone under various soaking water temperatures. The dimensions of the NDB specimen made of sandstone are 80 mm in length, 40 mm in width, 32 mm in thickness, with a 16 mm crack length. The span between the two supporting points at the bottom is 40 mm, and the applied load is *P*. The parameters of the NDB specimen made of sandstone are set as *d/W* = 0.5, *a/W* = 0.4, achieving pure mode I and mode II fracture toughness tests at two different crack inclination angles of *α* = 0° and 45.9°, respectively^45^.

The stress intensity factors *K*_I_, *K*_II_ and *T*-stress in the NDB specimen can be calculated using the following formula, based on the geometric parameters and the applied load.

1 $$K_{{\text{I}}} = \frac{{P\sqrt {\pi a} }}{2WB}Y_{{\text{I}}} \left( {\frac{a}{W},\frac{a}{W},\alpha } \right)$$

2 $$K_{{{\text{II}}}} = \frac{{P\sqrt {\pi a} }}{2WB}Y_{{{\text{II}}}} \left( {\frac{a}{W},\frac{a}{W},\alpha } \right)$$

3 $$T = \frac{P}{2WB}T^{*} \left( {\frac{a}{W},\frac{a}{W},\alpha } \right)$$

where *Y*_I_ and *Y*_II_ are the mode I and mode II nondimensional stress intensity factors, respectively; T^*^ is the nondimensional form of the *T*-stress.

### Numerical simulation of the fracture parameters of NDB specimens

**Figure 2:**
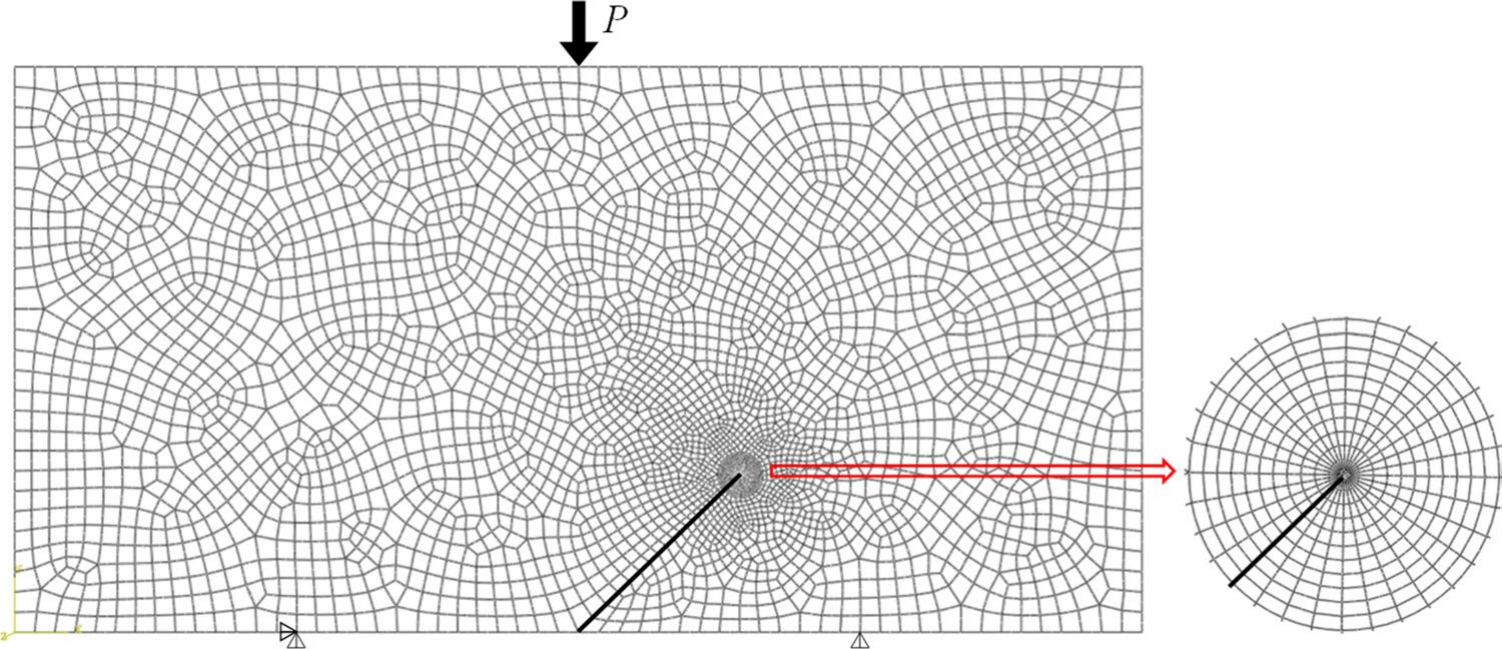
Typical finite element mesh division of an NDB specimen.

To calculate the dimensionless parameters *Y*_I_, *Y*_II_ and T^*^, several numerical models of the NDB specimen were established using the finite element model (FEM) code Abaqus. The elastic modulus and Poisson's ratio were set to 20 GPa and 0.21, respectively, assuming linear elastic behavior for the material. The length *L*, width *W*, and thickness *B* of each NDB specimen were set to 80, 40, and 32 mm, respectively. As shown in Fig. 2, the displacements in the *Y* direction at the two bottom supports were constrained to zero, and the displacement in the *X* direction at the left bottom support was also fixed. Due to the stress field singularity around the crack tip, singular elements were used in the first ring around the crack tip in the finite element model. The size of these singular elements was less than 1/100th of the crack length. To ensure a smooth curve of the *J*-integral paths, 11 rings of quadrilateral elements were meshed around the singular elements using the sweep technique. The outer edge of the outermost ring was approximately 1/9th of the crack length from the crack tip. The rest of the model was meshed with freely divided quadrilateral elements. The model consisted of approximately 3500 8-node biquadratic plane strain quadrilateral elements (CPE8).

**Figure 3:**
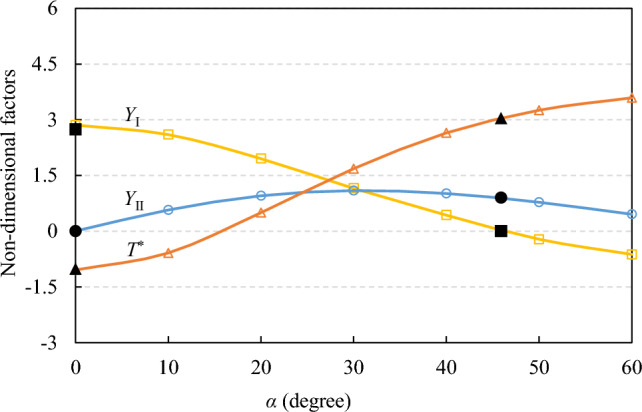
Variations of geometry factors for different values of the inclined angle *α*; the open point data were obtained by Luo et al.^45^, and the solid point data were obtained from this study.

Applying a unit load *P* = 1 N, the dimensionless geometric parameters *Y*_I_, *Y*_II_ and T^*^ for two different crack inclination angles of *α* = 0° and 45.9° were calculated using formulas (1) to (3) after obtaining *T*-stress, *K*_I_ and *K*_II_ through numerical computation. When *α* = 0°, the calculated values of *Y*_I_ and T^*^ are 2.739 and − 1.018; when *α* = 45.9°, the calculated values of *Y*_II_ and T^*^ are 0.8996 and 3.041, respectively. To verify the accuracy of the numerical analysis results, the values (solid points) were compared with the results calculated by Luo et al.^45^ (the void points), as shown in Fig. 3. An excellent concordance between both results is observable.

### Specimens preparation and testing process

**Figure 4:**
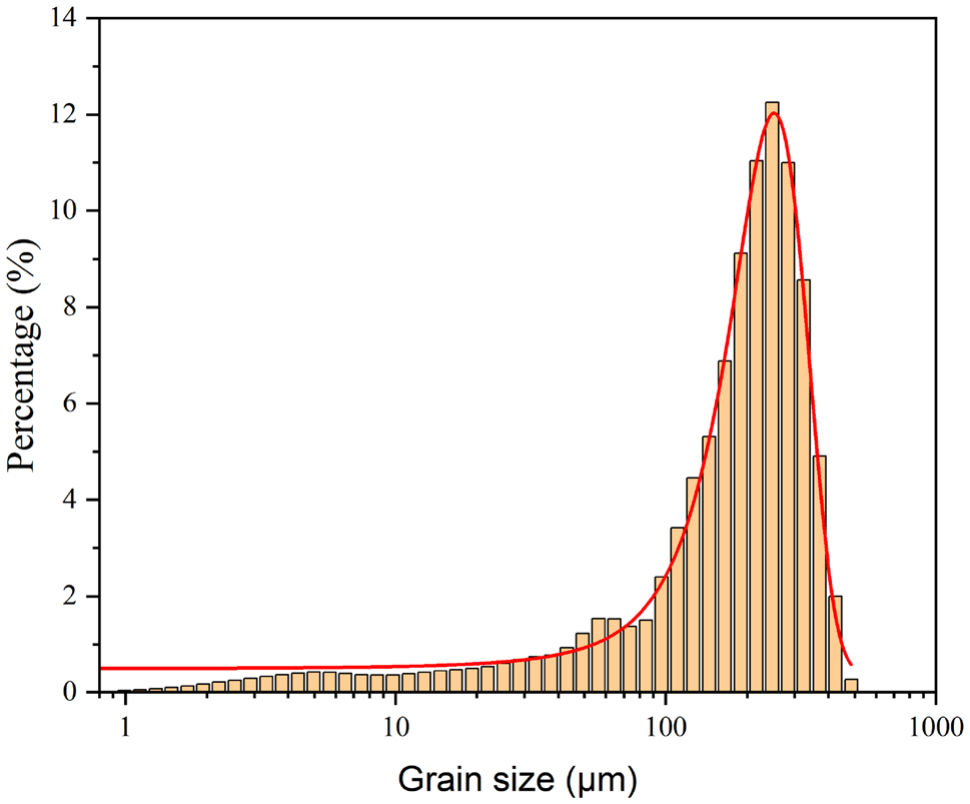
Particle size distribution for the tested sandstone.

The sandstone used in this experiment was obtained from the sandstone outcrops in Longchang, Sichuan Province. Visual inspections revealed that the tested material, a typical sandstone, was continuous, homogeneous, and gray in color. The mineral species and contents in the tested sandstone were obtained via X-ray diffraction (XRD) analysis, which indicated that the sandstone is composed of kaolinite, albite, chlorite, low quartz, and muscovite. The percentages of each mineral are shown in Table 1. The particle size distribution of the sandstone, as depicted in Fig. 4, was detected using a laser particle size analyzer. This analysis delineated a relatively concentrated distribution of sandstone grains within the size range of 100 to 400 µm.

**Table 1:** Mineral content of the tested sandstone.

Mineral composition	Kaolinite	Albite	Chlorite	Low Quartz	Muscovite
Content (%)	6.6	34.0	10.3	46.7	2.4

**Figure 5:**
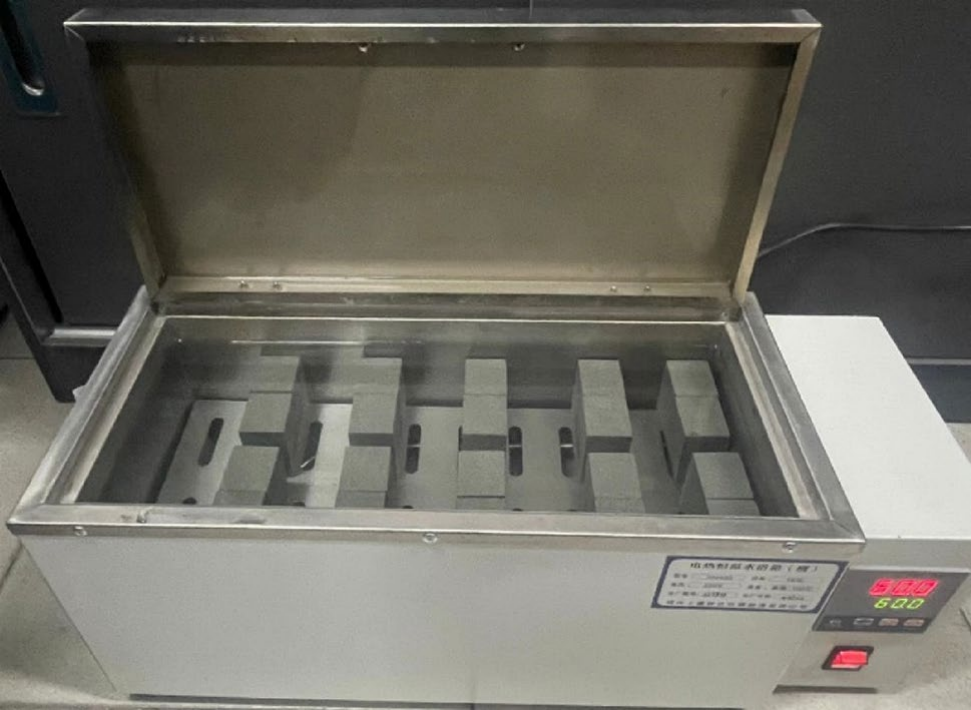
Specimens subjected to soaking at the constant-temperature water.

These specimens, with crack angles of *α* = 0° and 45.9°, were used to measure the pure mode I and pure mode II fracture toughness of sandstone. The experimental procedure was as follows: sandstone samples with the same crack angle were divided into four groups. To measure the water content of sandstone samples, they were first subjected to a standard drying process before the water bath. During this drying process, the samples were placed in a drying oven at 110 °C for 24 h. After drying, the samples were removed and weighed. The four groups were then placed respectively in a dry room-temperature environment and constant-temperature water baths at 23 °C, 60 °C, and 99 °C. The water baths were preheated to the desired temperatures, and then each group of specimens was fully immersed in the corresponding water bath for 48 h (see Fig. 5). The specimens were then weighed again after the water bath. The increase in weight was used to determine the water content of the specimen:

4 $$\omega_{w} = \frac{{m_{w} - m_{d} }}{{m_{d} }} \times 100\%$$

where *ω*_*w*_ is the water content of the specimen, *m*_*w*_ and *m*_*d*_ are the wet and dry masses of the specimen, respectively. The average water contents were measured as 3.9%, 3.6%, and 3.4% for samples soaked at 23 °C, 60 °C, and 99 °C, respectively. The specimens exhibited a gradual decline in average water content as the soaking temperature increases.

**Figure 6:**
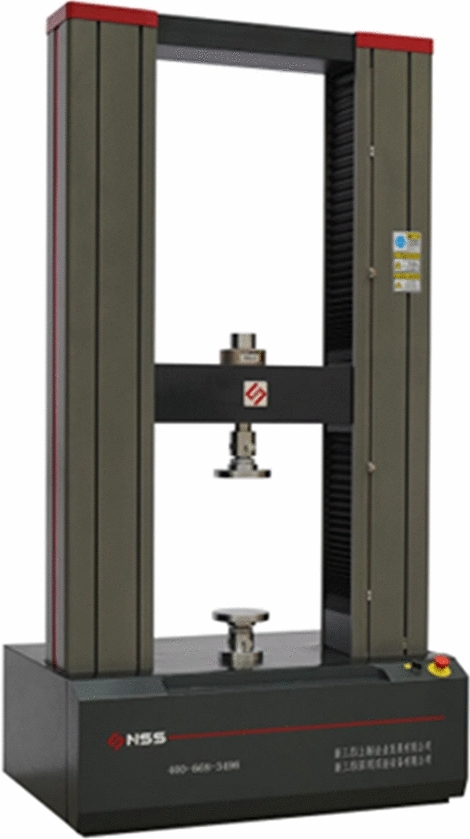
5T universal testing machine.

**Figure 7:**
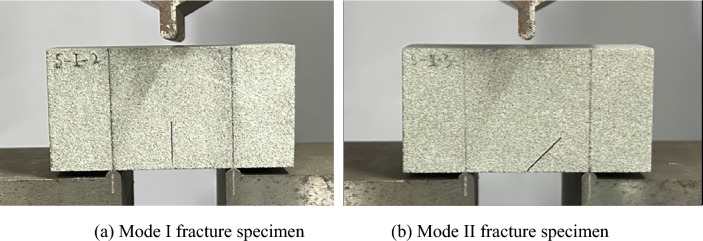
Loading device and NDB specimens.

After removal from the baths, the samples were immediately tested in a universal testing machine (Fig. 6). This microcomputer-controlled electronic universal testing machine is designed to handle a maximum test force of 5 T. It is equipped with an all-digital AC servo control system that incorporates a high-precision and high-response frequency AC servo motor. This setup ensures efficient and balanced transmission, while maintaining a speed error strictly within ± 0.2%. The NDB specimens and loading device for the mode I and mode II fracture toughness tests are illustrated in Fig. 7. During the fracture tests, static loading was consistently applied at a slow, uniform displacement loading rate of 0.02 mm/min.

## Experimental results

### Load–displacement curves and failure patterns

**Figure 8:**
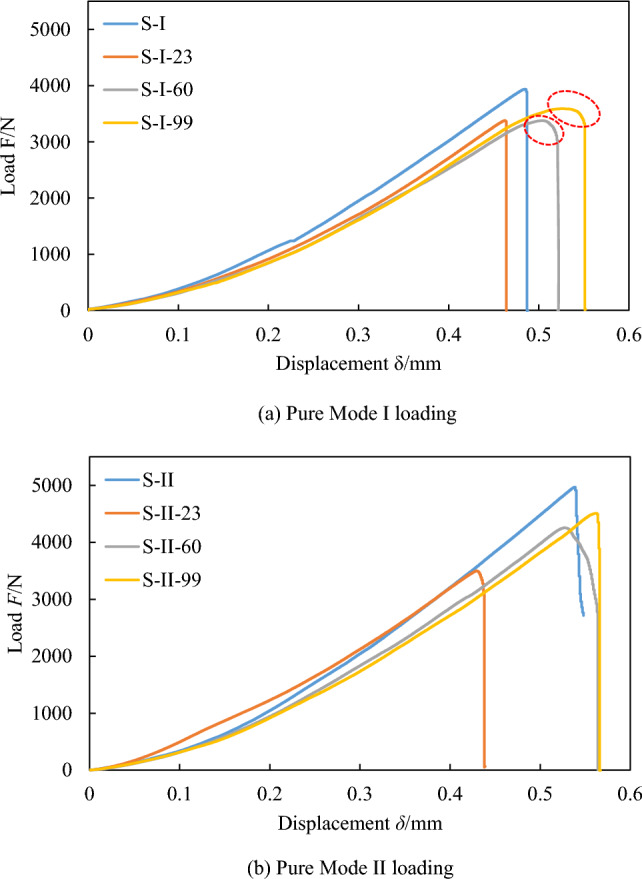
Load–displacement curves of sandstone fracture tests subjected to soaking at different water temperatures.

Figure 8 depicts the load–displacement curves for pure mode I and pure mode II fracture tests on sandstone after soaking in water at various temperatures. It is apparent that the general trends of these curves in both fracture modes maintain approximately consistent behavior. During the initial loading phase, the load–displacement curves exhibit a concave shape, primarily a result of microcrack closure within the specimen due to the applied load. In the subsequent elastic phase, the curve becomes more linear, rising to its peak value. As evident in Fig. 8, the slope of the elastic portion of the load–displacement curves for the water-soaked sandstone specimens is less steep compared to those of the dry specimens, indicating a reduction in stiffness. This is largely due to the growth and expansion of microcracks within the rock from water soaking, leading to increased softening of the sandstone. As the soaking temperature increases, the softening effect intensifies, resulting in a steeper decrease in slope. The maximum deformation, or displacement at peak load, appears to be greater for water-soaked specimens, especially for mode I specimens. For example, the increase in the maximum deformation for mode I specimens soaked at 99 °C was measured to be 0.064 mm compared to dry specimens, which suggests enhanced ductility under coupled water and thermal treatments.

**Figure 9:**
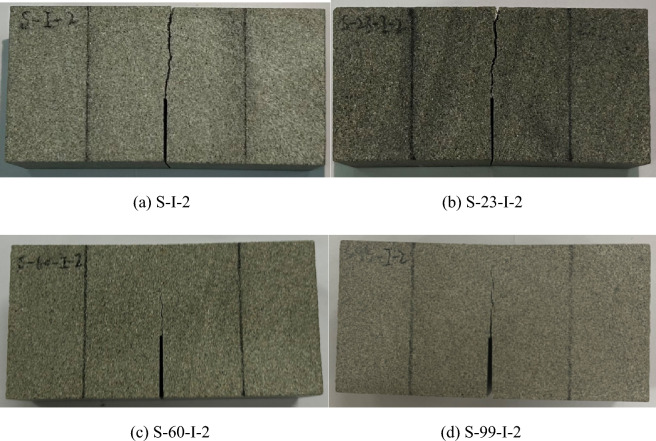
Photos of sandstone mode I fracture specimens subjected to soaking at different water temperatures.

**Figure 10:**
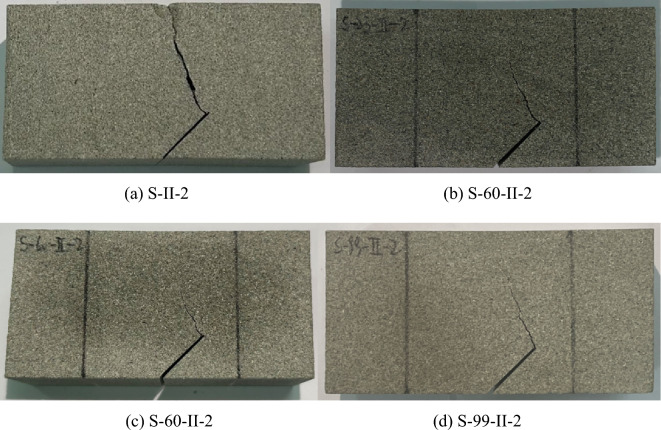
Photos of sandstone mode II fracture specimens subjected to soaking at different water temperatures.

Figures 9 and 10 display typical post-failure photos of mode I and mode II fracture test specimens subjected to soaking at different water temperatures. The crack propagation in all specimens initiates from the prefabricated crack tip and extends towards the top loading point. In mode I specimens, the crack path is almost straight, while in mode II specimen, it forms a concave curve. A close examination of the post-failure photos reveals that the mode II fracture specimens not subjected to soaking split entirely along the crack path. By contrast, those subjected to soaking did not separate into two independent parts by the end of the loading process. In the mode I fracture specimens, both unsoaked and 23 °C water-soaked specimens split into two independent parts along the crack path, demonstrating the brittle fracture characteristic of sandstone, as evidenced by the sudden unloading following the peak load of the load–displacement curves in Fig. 8a. However, specimens water-soaked at higher temperatures (60 °C, 99 °C) did not split entirely along the crack path, with the load–displacement curve's peak exhibiting a curved shape, as highlighted with red circles in Fig. 8a. This indicates that with increasing soaking water temperatures, the transition in sandstone's failure characteristics from brittle to ductile becomes more evident. This is because the high temperature causes the thermal expansion of mineral crystals in sandstone specimens, resulting in compression deformation of internal grain gaps and the closure of many original and newly generated microcracks, leading to ductility characteristics during crack propagation. Meanwhile, a clear difference in post-peak behavior was observed between the mode I and mode II specimens in Fig. 8, particularly noting increased ductility in the S-I-60 and S-I-99 specimens subjected to higher soaking temperatures, while the ductile behavior is less pronounced in the S-II-60 and S-II-99 specimens. Alneasan and Alzo’ubi et al.^39,49^ investigated the effect of thermal treatment on fracture behavior of granite and mudstone under three loading modes (I, I/II, and II), and found that increasing the mode mixity ratio from pure mode I to mixed mode loading I/II and pure mode II under the same temperature enhances rock brittle behavior, which is consistent with the observation in the present study.

### The influence of coupled water and thermal treatments on the mode I and mode II fracture toughness of sandstone

By substituting the peak loads obtained from the experiments into the stress intensity factor Eqs. (1)–(2) formulated for NDB specimens, the fracture toughness of sandstone can be calculated. The results of mode I and mode II fracture toughness tests on sandstone subjected to soaking in water at various temperatures are comprehensively presented in Tables 2 and 3. In both the pure mode I and mode II fracture tests, the peak loads observed in the sandstone specimens subjected to soaking at various temperatures are significantly lower than those in the unsoaked specimens. This reduction in strength is ascribed to water's solvent effect, which lessens the cohesion between the mineral particles inside the rock during soaking, leading to the weakening of the rock's internal structure. The degradation degree of the mode I and mode II fracture toughness as a result of soaking at different water temperatures, denoted as *S*_I_ and *S*_II_, can be determined by applying the following formulas:

5 $$S_{{\text{I}}} = \frac{{K_{{{\text{IC}}}} - K_{{{\text{IC}}j}} }}{{K_{{{\text{IC}}}} }} \times 100\% ,\;S_{{{\text{II}}}} = \frac{{K_{{{\text{IIC}}}} - K_{{{\text{IIC}}j}} }}{{K_{{{\text{IIC}}}} }} \times 100\% ,\;\left( {j = 23,60,99} \right)$$

where *K*_IC_ and *K*_IIC_ represent the average values of the mode I and mode II fracture toughness of unsoaked sandstone, respectively. *K*_IC*j*_ and *K*_IIC*j*_ are the average values for mode I and mode II fracture toughness of sandstone after soaking at *j* °C. As observed in Tables 2 and 3, the average *K*_IC_ value for dry sandstone is 0.9474 MPa·m^0.5^. After soaking at 23 °C, 60 °C, and 99 °C, the average values of mode I fracture toughness decrease by 15.4%, 14.3%, and 13.2%, respectively. Comparatively, the mode II fracture toughness of sandstone is lower than that of mode I under identical conditions. The average *K*_IIC_ value for dry sandstone is 0.3908 MPa·m^0.5^, exhibiting reductions of 26.1%, 21.2%, and 8.9% respectively after soaking at 23 °C, 60 °C, and 99 °C. This indicates that coupled water and thermal treatments have a certain deteriorating effect on both mode I and mode II fracture toughness of sandstone. Additionally, the soaking effect causes greater deterioration in *K*_IIC_ than in *K*_IC_ at low water temperatures.

**Table 2:** Results of Mode I fracture tests on sandstone subjected to soaking at different water temperatures.

Specimen no	Treatment conditions	Peak load* P*cr (kN)	Mode I fracture toughness (MPa·m^0.5^)		The degradation degree *S*_I_ (%)
			Test data	Average value	
S-I-1	Unsoaked	4.084	0.9795	0.9474	
S-I-2	Unsoaked	3.83	0.9186		
S-I-3	Unsoaked	3.936	0.9440		
S-23-I-1	Soaking in 23 °C water	3.323	0.7970	0.8013	15.4
S-23-I-2	Soaking in 23 °C water	3.32	0.7963		
S-23-I-3	Soaking in 23 °C water	3.38	0.8107		
S-60-I-1	Soaking in 60 °C water	3.374	0.8092	0.8119	14.3
S-60-I-2	Soaking in 60 °C water	3.405	0.8167		
S-60-I-3	Soaking in 60 °C water	3.376	0.8097		
S-99-I-1	Soaking in 99 °C water	3.344	0.8020	0.8219	13.2
S-99-I-2	Soaking in 99 °C water	3.591	0.8613		
S-99-I-3	Soaking in 99 °C water	3.345	0.8023

**Table 3:** Results of Mode II fracture tests on sandstone subjected to soaking at different water temperatures.

Specimen no	Treatment conditions	Peak load* P*cr (kN)	Mode II fracture toughness (MPa·m^0.5^)		The degradation degree* S*_II_ (%)
			Test data	Average value	
S-II-1	Unsoaked	4.929	0.3883	0.3908	
S-II-2	Unsoaked	4.972	0.3917		
S-II-3	Unsoaked	4.98	0.3924		
S-23-II-1	Soaking in 23 °C water	3.327	0.2621	0.2887	26.1
S-23-II-2	Soaking in 23 °C water	4.155	0.3274		
S-23-II-3	Soaking in 23 °C water	3.509	0.2765		
S-60-II-1	Soaking in 60 °C water	3.445	0.2714	0.3079	21.2
S-60-II-2	Soaking in 60 °C water	4.267	0.3362		
S-60-II-2	Soaking in 60 °C water	4.472	0.3523		
S-60-II-4	Soaking in 60 °C water	3.449	0.2717		
S-99-II-1	Soaking in 99 °C water	4.357	0.3433	0.3561	8.9
S-99-II-2	Soaking in 99 °C water	4.52	0.3561		
S-99-II-3	Soaking in 99 °C water	4.683	0.3690		

**Figure 11:**
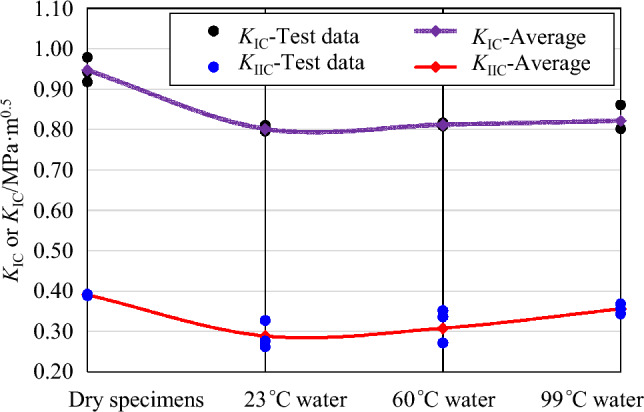
Mode I and mode II fracture toughness of sandstone subjected to soaking at different water temperatures.

Figure 11 illustrates the trends in mode I and mode II fracture toughness of sandstone subjected to soaking in water at different temperatures. It is evident that both mode I and mode II fracture toughness values decrease significantly after soaking at various water temperatures. This decrease in fracture toughness occurs because after soaking treatment, water molecules enter the microcracks and pores inside the specimen. As a solvent, water causes some minerals in the sandstone to dissolve and leach, which leads to a decrease in cohesion between mineral particles and the propagation of microcracks in the rock, thus reducing the ability of sandstone specimens to resist crack propagation. Within the studied temperature range of 23 °C to 99 °C, the mode I and mode II fracture toughnesses of sandstone show a slightly increasing trend with rising water temperature, although the trend for mode I fracture toughness is not as obvious. This indicates that mode II fracture toughness of sandstone is more sensitive to the soaking water temperature.

## Theoretical analysis of fracture strength

The Maximum Tangential Stress (MTS) criterion proposed by Erdogan and Sih^50^ assumes that a crack propagates in the direction *θ*_0_ on which the tangential stress *σ*_*θθ*_ around the crack tip reaches its maximum, and the crack initiation begins when the maximum tangential stress reaches a critical value *σ*_*θθc*_, i.e.,

6 $$\frac{{\partial \sigma_{\theta \theta } }}{\partial \theta } = 0,\;\frac{{\partial^{2} \sigma_{\theta \theta } }}{{\partial \theta^{2} }} < 0\;at\;\theta = \theta_{0} ,\;\sigma_{\theta \theta c} = \frac{{K_{{{\text{IC}}}} }}{{\sqrt {2\pi r_{0} } }}$$

where *r*_0_, known as the fracture process zone (FPZ) radius, is the distance from the crack tip to the boundary of the core region along the crack propagation direction. The tangential stress *σ*_*θθ*_ around the crack tip can be calculated using the following equations:

7 $$\sigma_{\theta \theta } = \frac{1}{{\sqrt {2\uppi r} }}\cos \frac{\theta }{2}\left( {K_{\rm I} \cos^{2} \frac{\theta }{2} - \frac{3}{2}K_{{{\text{II}}}} \sin \theta } \right)$$

Regardless of *T*-stress and assuming *r*_0_ as a material constant, The MTS criterion yields initial crack angles and critical loads that are independent of *r*_0_. Smith et al.^51^ introduced *T*-stress into the MTS criterion, formulating the Generalized Maximum Tangential Stress (GMTS) criterion. In the GMTS criterion, *r*_0_ is assumed to be a material constant and can be calculated using the following formula

8 $$r_{0} = \frac{1}{2\pi }\left( {\frac{{K_{{{\text{IC}}}} }}{{\sigma_{t} }}} \right)^{2} .$$

where *K*_IC_ and *σ*_*t*_ represent the mode I fracture toughness and the tensile strength of sandstone after soaking in water at different temperatures, respectively. The tensile strengths of the dry sandstone and those soaked at 23 °C, 60 °C, and 99 °C for 48 h are 4.045 MPa, 3.720 MPa, 3.841 MPa and 4.064 MPa respectively, which were measured using the Brazilian splitting test. The comparable tension strength between dry specimens and water-soaked specimens at high temperature (99 °C) could be due to several factors. Firstly, the thermal treatment may cause mineral grains to expand and exert compressive forces within the grain boundaries, effectively closing off microcracks and reducing their detrimental effect on tensile strength. Secondly, although water weakens the rock matrix, the elevated temperature might lead to partial dehydration and consolidation of the rock, which could mitigate the loss of strength normally associated with water saturation. Substituting these tensile strength values into Eq. (7) calculates the FPZ radii for the dry sandstone and those soaked at various temperatures, resulting in radii values of 8.728 mm, 7.382 mm, 7.110 mm and 6.509 mm respectively. This indicates that water soaking significantly reduces the FPZ radius in sandstone, with the radius gradually decreasing as the water temperature increases.

**Figure 12:**
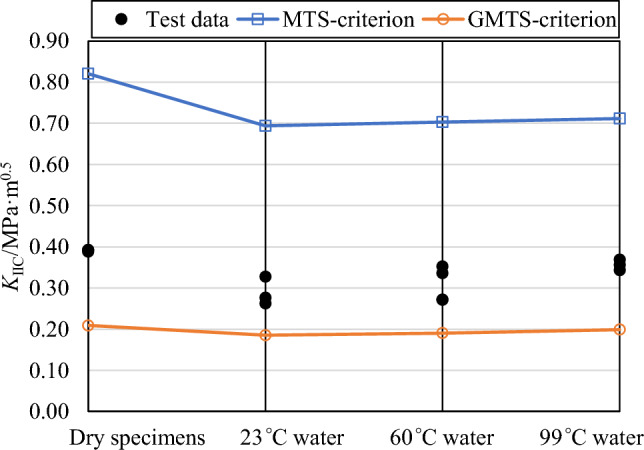
Mode II fracture toughness predicted by the GMTS criterion and the MTS criterion.

Based on the GMTS criterion, the initial crack initiation angle in pure mode II fracture mode and the relationship between mode I and mode II fracture toughness for NDB specimens can be further determine:

9 $$3\cos \theta_{0} - 1 - \frac{16}{3}\sin \frac{{\theta_{0} }}{2}\cos \theta_{0} \sqrt {\frac{{2r_{0} }}{a}} \frac{{T^{*} }}{{Y_{{{\text{II}}}} }} = 0$$

10 $$\frac{{K_{{{\text{IIC}}}} }}{{K_{{{\text{IC}}}} }} = \left( { - \frac{3}{2}\sin \theta_{0} \cos \frac{{\theta_{0} }}{2} + \frac{{T^{*} }}{{Y_{{{\text{II}}}} }}\sqrt {\frac{{2r_{0} }}{a}} \sin^{2} \theta_{0} } \right)^{ - 1} .$$

where *θ*_0_ is the initial crack initiation angle in pure mode II fracture mode for NDB specimens. By substituting the mode I fracture toughness *K*_IC_ and FPZ radius *r*_0_ of sandstone subjected to soaking at various temperatures into Eqs. (9) and (10), the mode II fracture toughness predicted by the GMTS criterion can be calculated, as shown in Fig. 12. This figure compares the predicted mode II fracture toughness values of the MTS and GMTS criteria for both unsoaked and water-soaked sandstone at three different temperatures with the experimental results. It clearly reveals that the MTS criterion significantly overestimates the mode II fracture toughness compared to the experimental findings in all four treatment conditions. The predictions of the GMTS criterion are slightly lower than the experimental values, showing a smaller error than those of the MTS criterion. This indicates that the GMTS criterion, which considers *T*-stress, more accurately predicts the mode II fracture toughness of sandstone subjected to soaking at different water temperatures than the MTS criterion.

**Figure 13:**
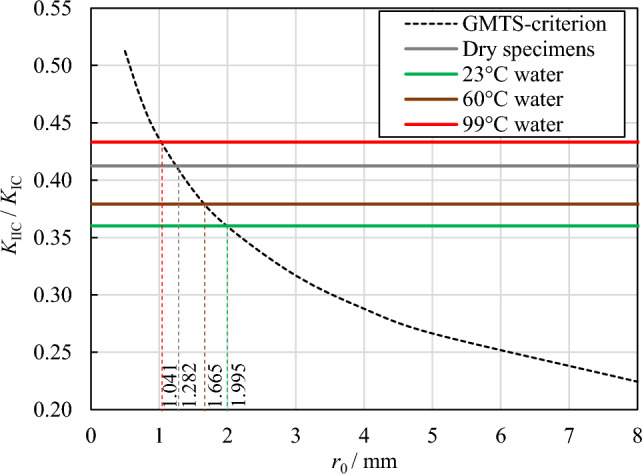
Prediction of the fracture process zone radius *r*_0_ in the sandstone at different soaking temperature conditions using the GMTS criteria.

Figure 12 shows that the predictions of both the MTS and GMTS criteria deviate from the experimental results of mode II fracture toughness in sandstone under various conditions. A possible reason for this deviation is that both criteria assume the FPZ radius *r*_0_ as a material constant independent of the loading mode. For example, *r*_0_ used in the GMTS criteria is calculated using Eq. (8) based on the fracture toughness under the pure mode I loading condition. This assumption appears questionable as other scholars have emphasized that the FPZ size should not remain constant^32,39,52^. Consequently, within the scope of this study, the FPZ radius *r*_0_ was determined based on the ratio of mode II to mode I fracture toughness (*K*_IIC_/*K*_IC_). To estimate *r*_0_, the average value of *K*_IIC_/*K*_IC_ experimentally measured were plotted independently of *r*_0_ and presented as distinct horizontal lines in Fig. 13, each representing a specific soaking water temperature condition. Subsequently, the analytical prediction of *K*_IIC_/*K*_IC_ was conducted utilizing the GMTS criteria and depicted in Fig. 13 as a function of *r*_0_. The value of *r*_0_ was ascertained at the point of the intersection between the analytical and experimental fracture toughness ratio, as illustrated in Fig. 13. It is evident from the figure that the GMTS criteria successfully predicted the experimental *K*_IIC_/*K*_IC_ of the dry sandstone and those soaked at 23 °C, 60 °C, and 99 °C for 48 h at *r*_0_ = 1.282, 1.665, 1.995, and 1.041 mm, respectively. Namely, the *r*_0_ was predicted using the GMTS criteria in the sandstone at different soaking temperature conditions. Notably, the *r*_0_ derived from the fracture toughness ratio *K*_IIC_/*K*_IC_ is significantly lower than that calculated from mode I fracture toughness *K*_IC_ using Eq. (8). Furthermore, with an increase in soaking water temperature, the *r*_0_ value calculated from Eq. (8) gradually decreases, while the* r*_0_ calculated from *K*_IIC_/*K*_IC_ exhibited an opposite trend of increase. Therefore, the previous assumption that the fracture process zone radius *r*_0_ is a constant, independent of the loading mode, does not fully align with experimental results. Further research about the fracture process zone is needed to understand the mechanisms involved more deeply.

## Conclusions

This study employs NDB specimens to investigate the influence of coupled water and thermal treatments at various temperatures (23 °C, 60 °C, and 99 °C) on mode I and mode II fracture characteristics of sandstone. It also discusses the predicted values of mode II fracture toughness and the fracture process zone based on the Maximum Tangential Stress criterion. The main research conclusions are summarized as follows:

1. Under soaking at various water temperatures, both mode I and mode II fracture toughness of sandstone significantly decrease. Within the temperature range studied, the mode I and mode II fracture toughnesses of sandstone demonstrate a slightly rising trend with increasing water temperatures, with reductions ranging from 15.4% to 13.2% for mode I and 26.1% to 8.9% for mode II respectively.
2. When soaked at lower water temperatures, the fracture process of sandstone demonstrates typical characteristics of brittle fracture. With an increase in the soaking water temperature, mode I specimens gradually transition from brittle to ductile, while the ductile behavior is less pronounced in mode II specimens.
3. Both the GMTS and MTS criteria have discrepancies to in predicting the mode II fracture toughness of sandstone subjected to soaking at different water temperatures. However, the GMTS criterion, which accounts for *T*-stress, demonstrates a smaller margin of error relative to the MTS criterion. The conventional assumption that the fracture process zone radius *r*_0_ remains constant and independent of the loading mode lacks evidence and requires further investigation through theoretical and experimental data.

## Data Availability

All data generated or analyzed during this study are included in this published article.
